# The Role of Artificial Intelligence in Female Infertility Diagnosis: An Update

**DOI:** 10.3390/jcm14093127

**Published:** 2025-04-30

**Authors:** Necati Findikli, Catherine Houba, David Pening, Anne Delbaere

**Affiliations:** 1 Fertility Clinic, Department of Obstetrics and Gynecology, Université Libre de Bruxelles (ULB), Hôpital Universitaire de Bruxelles (H.U.B), CUB Hôpital Erasme, Route de Lennik 808, 1070 Bruxelles, Belgium; catherine.houba@hubruxelles.be (C.H.); david.pening@hubruxelles.be (D.P.); anne.delbaere@hubruxelles.be (A.D.); 2 Research Laboratory on Human Reproduction, Université Libre de Bruxelles (ULB), Hôpital Universitaire de Bruxelles (H.U.B), CUB Hôpital Erasme, Route de Lennik 808, 1070 Bruxelles, Belgium

**Keywords:** artificial intelligence, female infertility, reproductive medicine

## Abstract

Female infertility is a multifaceted condition affecting millions of women worldwide, with causes ranging from hormonal imbalances and genetic predispositions to lifestyle and environmental factors. Traditional diagnostic approaches, such as hormonal assays, ultrasound imaging, and genetic testing, often require extensive time, resources, and expert interpretation. In recent years, artificial intelligence (AI) has emerged as a transformative tool in the field of reproductive medicine, offering advanced capabilities for improving the accuracy, efficiency, and personalization of infertility diagnosis and treatment. AI technologies demonstrate significant potential in analyzing vast and complex datasets, identifying hidden patterns, and providing data-driven insights that enhance clinical decision-making processes in assisted reproductive technologies (ART) services. This narrative review explores the current advancements in AI applications in female infertility diagnostics and therapeutics, highlighting key technological innovations, their clinical implications, and existing limitations. It also discusses the future potential of AI in revolutionizing reproductive healthcare. As AI-based technologies continue to evolve, their integration into reproductive medicine is expected to pave the way for more accessible, cost-effective, and personalized fertility care.

## 1. Introduction

Infertility, which is clinically defined as the inability to achieve a clinical pregnancy after 12 months of regular and unprotected sexual intercourse, stems from a myriad of factors associated with the male, the female, or in certain cases, both. Also, in 10–25% of the cases, it remains unexplained despite thorough investigation [1]. Female infertility accounts for approximately 40% of all infertility cases and can result from various factors, including hormonal imbalances, structural abnormalities, genetic predispositions, and lifestyle-related issues [2]. The ability to correctly diagnose the cause(s) of infertility is the key to seeking or developing effective treatment strategies that address the underlying factors and optimize reproductive outcomes, and failure to do so results in ineffective treatment approaches, unnecessary interventions, emotional distress, and financial burdens for patients [3].

Modern diagnostic tools, such as hormonal assessments, imaging techniques, and genetic testing, provide valuable insights into the reproductive health status of an individual. Effective diagnosis of infertility-associated conditions or factors can allow fertility specialists to use targeted therapies such as surgical interventions, medical therapies, or assisted reproductive technologies (ART). Furthermore, precise diagnoses would facilitate personalized treatment approaches, improving the likelihood of conception while minimizing potential complications and side effects [4,5].

Technological advancements in recent years have created a considerable increase in the volume and complexity of biomedical data in medicine. While it paves the way for new opportunities in terms of diagnosis and treatment, it also poses numerous challenges [6]. Especially in the last two decades, there has been a continuous and considerable transformation in the diagnosis, management, and treatment of infertility thanks to the advances in molecular biology and genetics coupled with translational bioinformatics. That is, the development of methods to store, analyze, and interpret vast amounts of OMICs data into predictive and therapeutic applications has nowadays been increasingly investigated in the infertility diagnosis [7,8,9]. Such a transformation also necessitates not only high computation power but also novel approaches to uncover underlying pathology, highlighting the need for personalized diagnostic and therapeutic approaches to address the diverse etiologies of female infertility effectively [10,11,12].

AI functions by simulating human intelligence through algorithms and computational models that process large amounts of data, learn from it, and make decisions or predictions based on patterns and trends. Thanks to the availability of large datasets and improvements in computing power, there has been a worldwide and exponential growth of AI-related advancements over the past few years, leading to a transition from the experimental to the implementation phase in various fields of healthcare, including reproductive medicine [13].

In this narrative review, we aim to summarize the current stage of AI utilization in the context of female infertility diagnosis, expected areas of improvement, and potential and unforeseen risks/disadvantages that would come with such an innovation in the diagnosis as well as the management of female infertility.

## 2. Methodology

A search query in the PubMed, EMBASE, and Scopus databases performed within the time frame of January 2010 and December 2024 by using the query “Artificial Intelligence” AND “Female infertility” retrieved 38, 73, and 47 studies, respectively. Records were further refined by eliminating the duplicated records as well as records that were not relevant to the study of interest. Original research papers, narrative reviews, systematic reviews, and meta-analyses were included, while editorials, conference abstracts, and technical articles were excluded. At the final stage, 44 studies were evaluated and included in the manuscript. References that were not present in the query results but considered important for the current work were also manually included by the authors.

## 3. Epidemiology and Pathophysiology of Female Infertility

Evaluation and diagnosis of female infertility are inherently complex due to the multifaceted interplay of genetic, hormonal, environmental, and lifestyle factors that contribute to reproductive challenges. It affects an estimated 8–12% of women of reproductive age globally, and its prevalence varies based on geographical, socioeconomic, and cultural factors. Causes of female infertility include ovulatory disorders (25–30%), tubal factors (20–25%), uterine abnormalities (10–15%), and unexplained infertility (10–20%). Understanding its distribution requires careful consideration of risk factors such as age, obesity, stress, and exposure to different lifestyle conditions as well as environmental toxins, which can disrupt reproductive function [3]. Pathophysiologically, female infertility involves intricate mechanisms within the hypothalamic–pituitary–ovarian axis, uterine environment, and fallopian tube function, with highly prevalent conditions such as polycystic ovary syndrome (PCOS), endometriosis, and diminished ovarian reserve playing significant roles.

## 4. AI Algorithms/Approaches in Female Infertility

**Figure 1:**
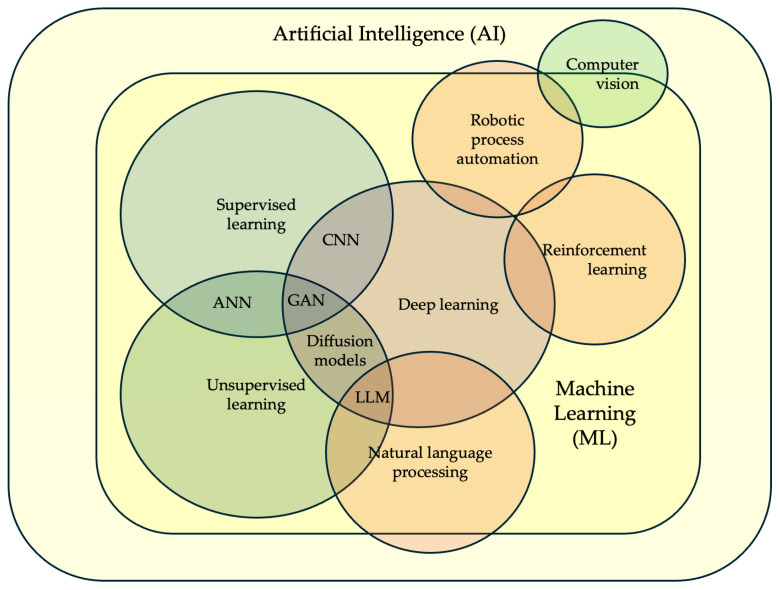
Types of AI algorithms commonly utilized in female infertility diagnosis and treatment. ANN: artificial neural networks, CNN: convolutional neural networks, GAN: generative adversarial networks, LLM: large language models. Adapted from [14].

Under the AI umbrella, machine learning (ML) algorithms (namely supervised, unsupervised, deep, and reinforcement learning frameworks) are nowadays widely used to analyze extensive datasets and identify patterns that predict the likelihood of a successful outcome in infertility diagnosis and treatment (Figure 1).

In supervised learning, algorithms infer functions by mapping inputs to outputs based on labeled training data, enabling the prediction or classification of unseen data. It excels when there is a clear understanding of the desired output for given inputs, making it suitable for tasks like image recognition and medical diagnosis. Similar to the structure of neurons in the brain, artificial neural networks (ANNs) are a network of interconnected computational units in a multilayered structure. The term “Deep learning (DL)” is used when ANNs with complex neuronal architectures with many layers form a convolutional neural network (CNN). They are usually very useful for spatial, grid-like data such as embryo images [14].

Unsupervised learning involves algorithms that learn patterns from unlabeled data, aiming to uncover hidden structures, relationships, or groupings without explicit guidance. Its primary goal is to discover inherent patterns, structures, or relationships within the data without relying on predefined labels. This approach is particularly useful for exploratory data analysis, where the aim is to gain insights into the underlying structure of the data. The most popular unsupervised algorithms are *k*-means, generative adversarial networks (GANs), and multimodal generative AI. Reinforcement learning is, on the other hand, a paradigm where an agent learns to make decisions in an environment to maximize a cumulative reward, learning through trial and error.

Unlike supervised learning, reinforcement learning does not rely on labeled data but rather learns from the consequences of its actions. The agent continuously interacts with the environment, observes the resulting state, and receives a reward or penalty based on its actions.

Natural language processing (NLP) is mainly used to extract and analyze vast amounts of textual data from existing digital patient records, scientific literature, and clinical guidelines, assisting healthcare providers in making informed decisions and tailoring treatment protocols [15]. The robotic process automation (RPA) approach is preferred to streamline administrative processes such as appointment scheduling, lab result reporting, and treatment tracking, enhancing efficiency and reducing human errors [16]. The computer vision approach, on the other hand, offers automated sperm and oocyte quality assessment by analyzing microscopic images and assessing sperm motility and morphology with greater accuracy and speed than traditional manual methods [17].

In general, while an AI model is generated, available data are divided into three sections: The “training” section is used to “train/teach” the model, the “validation” section is used to measure the performance and fine-tune the model, and the “test section” is used to independently test the model’s performance on “new” or “previously unknown” data. There also exist numerous additional strategies to improve the prediction outcome and prevent the “overfitting”, which is characterized by the successful performance of the model on the training data but poor performance on the test data.

The use of ML is very efficient on structured/tabular data with small to medium datasets, but it is less effective on unstructured data such as images, text, etc. Deep learning, on the other hand, is preferred when the data are unstructured. On the other hand, it requires a considerable amount of data to compute, and the results may not be very easily interpretable. In cases where text and language processing is needed, NLP becomes the algorithm of choice.

## 5. The Role of AI in Contemporary Female Infertility Diagnosis

The diagnostic process in female infertility typically includes a detailed medical history, physical examination, and a series of tests to evaluate hormonal balance, ovarian reserve, and the structural integrity of reproductive organs. Common assessments encompass blood tests for hormone levels, imaging studies like transvaginal ultrasound, and procedures such as hystero-salpingo-foam sonography to examine the uterine cavity and fallopian tube patency [18]. The efficiency of these diagnostic methods largely varies in terms of the quality and quantity of the services provided, as well as the proficiency of the healthcare professional who provides them. Despite an increased pace of advancements, challenges remain in achieving a prompt and accurate diagnosis, particularly for conditions like endometriosis, which often involve a prolonged diagnostic timeline [19]. 

Emerging diagnostic tools, including NLP applications, AI-coupled non-invasive tests, imaging techniques, and medical decision-support systems, hold promise for enhancing diagnostic and therapeutic efficiency as well as helping medical professionals to improve counseling by predicting the likelihood of infertility based on risk factors, such as age, lifestyle, medical history, etc. [20]. Table 1 summarizes the key findings of the recently published studies in which the potential implications of AI in female infertility diagnosis have been investigated.

Ovarian reserve, indicated by markers such as anti-Müllerian hormone (AMH) and antral follicle count (AFC), is very important to tailor treatments and predict success rates in most infertility treatments. Studies indicate that ovarian reserve assessments can be improved by analyzing such parameters with clinical, genetic, lifestyle, and environmental parameters with the help of ML algorithms [21,22].

**Table 1:** Summary of the studies in which the contribution of AI has been analyzed. CE: chronic endometritis, DNN: deep neural network, LR: logistic regression, SVM: support vector machine, DT: decision trees, GB: gradient-boosting, XGB: extreme gradient boosting, RF: random forest, NB: naïve Bayes, SGD: stochastic gradient descent, NN: neural network, k-NN: k-nearest neighbor, MLP: multilayered perceptron.

Cited Ref.	Study Design	Condition(s) Studied	# of Women/ Samples	Algorithm(s) Tested	Key Findings
Shanmugavadivel et al., 2024 [23]	Retrospective cohort study	PCOS	541	NR, NB, SVM	Support vector machine model achieved high accuracy of 94.44%.
Yu et al., 2024 [7]	Retrospective cohort study	POI	10	RF, Boruta	It is the first study to characterize the transcriptional profile of POI using third-generation ONT sequencing. Seven candidate genes were identified based on the intersection features of the RF and Boruta algorithm.
Zad et al., 2024 [24]	Retrospective cohort study	PCO	30,601	LR, SVM, GB, RF	Prediction of PCOS prior to clinical diagnosis in patients achieved an average AUC of ≥80%
Qu et al., 2024 [8]	Retrospective cohort study	PCO, DOR, POI, Endometriosis, RIF, RPL	257	LR, SGD, NN, GB, RF	Seven potential biomarkers were identified for the diagnosis of the diseases analyzed. The ML method effectively distinguished subtle differences in urine metabolite fingerprints.
Lee et al., 2024 [25]	Retrospective cohort study	Endometrial CD138+ plasma cells as a diagnostic biomarker for endometrial inflammation	193	CNN	The AI algorithm consistently and reliably distinguished CD138− and CD138+ cells, with total error rates of 6.32% and 3.23%, respectively.
Kitaya et al., 2024 [26]	Retrospective cohort study	Endometrial micropolyps in infertile women with CE	244	CNN	The sensitivity, specificity, accuracy, precision, and F1-score of the CNN model-aided diagnosis were 93.6%, 92.3%, 92.8%, 88.0%, and 0.907, respectively. Developed deep learning-based CNN was found to be capable of recognizing endometrial micropolyps and displayed potential for further development of the computer-aided diagnostic system for CE.
Diaz-Gimeno 2024 [27]	Prospective multicenter study	Risk of endometrial failure	281	SVM, RF	The endometrial failure risk (EFR) signature revealed a novel endometrial disruption, independent of endometrial luteal phase timing, present in 73.7% of patients. This EFR signature strati ed patients into 2 significantly distinct and clinically relevant prognosis pro les providing opportunities for personalized therapy.
Xiong et al., 2023 [28]	Retrospective cohort study	CE	248	XGB	The sensitivity, specificity, and accuracy rates of AI diagnostic system of CE were 100%, 83.3%, and 91.4%, respectively. Compared to the manual method, the AI detection and counting of plasma cells was consistent and much less time-consuming.
Dabi et al., 2023 [29]	Prospective cohort study	Endometriosis	200	RF	Feature selection method generated a signature of 34 miRNAs linked to endometriosis-associated infertility. After validation, the most accurate signature model had a sensitivity, specificity, and area under the curve of 100%.
Yu et al., 2022 [30]	Prospective cohort study	Premature ovarian failure (POF)	120	Mean shift algorithm	Doppler ultrasound based on the artificial intelligence segmentation algorithm has shown the functional status and hemodynamics of the patient’s ovaries. The ovarian artery parameters PI and RI can be used as specific indicators for evaluating the POF.
Suha and Islam, 2022 [31]	Retrospective cohort study	PCO	594	CNN, XGB	The proposed algorithm significantly enhanced the accuracy while also reducing training execution time comparing with the other existing ML based techniques.
Kangasniemi et. al., 2022 [32]	Case control study	PCO	91	CNN	Automated cell counting with a deep learning model performs well for the human endometrium. Differences in leukocyte counts were not observed between the whole PCOS population and controls. However, anovulatory women with PCOS presented with a higher number of CD68þ cells in the epithelium and fewer leukocytes in the stroma compared with the controls.
Jakubczyk et al., 2022 [9]	Prospective cohort study	Idiopathic female infertility	116	RF, DT, k-NN, DNN, SVM, XGB	Analyzed ML methods showed that classification accuracy for the original set was from 93.75% to 100% depending on the learning algorithm used.
Liu and Ren, 2021 [33]	Prospective cohort study	Tubal patency	30	CNN	The diagnostic rate of routine MRI examination of the fallopian tube and other parts of the uterus was lower than or equal to that of MR-HSG examination by CNN.

Ovulation tracking is essential for timing intercourse or ART. Wearable devices and mobile apps can analyze physiological signals, such as basal body temperature and hormonal fluctuations, to predict ovulation. Incorporating AI into such systems has recently been reported to improve prediction accuracy compared to conventional methods, enhancing patient outcomes [34,35].

Medical imaging is a cornerstone of infertility diagnosis and management [36]. In recent years, the integration of AI techniques into medical imaging approaches has created considerable improvements in terms of imaging quality [33]. AI algorithms have already demonstrated their improved ability to assist in detecting and monitoring follicular development as well as in assessing endometrial health and receptivity [26,28,31,37,38,39]. As a result, these advancements can improve diagnostic accuracy, enhance clinical decision making, and ultimately, optimize treatment outcomes for female infertility diagnosis [21,30,40].

Throughout infertility diagnosis, AI-based prediction models have the potential to be integrated with different data sources, such as patient histories, physical symptoms, laboratory test results, hormonal profiles, imaging results, genetic information, and lifestyle factors, to identify subtle patterns and correlations that may be overlooked by traditional diagnostic methods to provide a more comprehensive and accurate diagnosis [21,23]. By leveraging predictive models, ML shows the potential to assist in the early detection of infertility-related conditions such as polycystic ovary syndrome (PCOS), endometriosis, and diminished ovarian reserve, enabling personalized treatment plans tailored to individual patient profiles [8,23,24,25,29,32,41,42,43,44]. Coupled with AI, the third-generation sequencing results have recently been used to refine the transcription profiles, identify seven candidate biomarkers, and delineate the genetic contributions in the pathophysiology of primary ovarian insufficiency (POI) [7]. AI has also been employed to evaluate uterine pathologies, such as fibroids, polyps, and endometriosis, as well as endometrial failure risk [26,27].

ChatGPT, an NLP-based generative AI tool that has received considerable public and professional interest, has already started to show extraordinary abilities in gathering digital information to be effectively used in health decision-making processes, including female infertility diagnosis for diseases such as PCOS and POI [45,46,47,48].

## 6. Applications of AI in ART

In recent years, advanced statistics, together with AI-based modeling, have emerged as promising tools not only in diagnosis but also in predicting fertility potential, IVF laboratory outcomes, and personalizing infertility treatments [49,50,51,52,53]. Such models can simulate various treatment schemes to help in pre-treatment counseling, to optimize the treatment by prediction and optimization of the gonadotropin dosing for ovarian stimulation, optimal trigger day prediction, maximization of ovarian stimulation outcome, and oocyte as well as embryo selection with the highest implantation success [14,54,55,56,57,58,59]. The first prospective multicenter randomized clinical trial, where the outcome of a standard starting gonadotropin dose given either by a clinician or an AI-based tool (iDoser) is compared, is currently underway [60]. Recent models have also been shown to successfully integrate patient-specific data, including age, hormonal levels, and genetic/proteomic data, to enhance treatment efficacy and reduce cycle failure rates [61,62]. A recent study reported that implantation success can be predicted after frozen embryo transfers by integrating histological and molecular data [63].

Due to its considerable commercialization potential, interest in creating AI-based clinical or laboratory outcome prediction (fertilization, embryo/blastocyst development, euploidy, implantation potential, and live birth outcomes, etc.) or non-invasive gamete or embryo selection tools is exponentially growing in the ART clinics/laboratories worldwide [64,65,66,67,68,69,70,71,72,73]. Currently, available commercial AI-based embryo selection tools utilize static morphology images, morphogenetic-based images, or videos to predict the developmental potential of gametes and embryos, promising better results than experienced embryologists [16,74,75,76]. Current research nowadays expands towards novel AI-based embryo selection approaches that combine not only images/videos but also all the data from the entire treatment cycle, alleviating the limits of clinical embryology practice for better prediction outcomes [61].

AI-driven innovations in wearable devices and mobile applications such as chatbots and virtual assistants also show considerable potential to allow patients to monitor their reproductive health in real time, facilitating early intervention and proactive management. As AI and AI-based technologies continue to evolve, they hold the potential to revolutionize ART by making fertility care more precise, efficient, and accessible.

## 7. Challenges and Limitations

Like any other recent innovation in the field of human reproduction, the scientific community has nowadays been witnessing an exponentially increased number of AI-based content in the literature, nearly all of which indicates novel algorithms with high predictive power or accuracy. Although they have a high potential to be commercialized, due to the nature of clinical as well as laboratory heterogeneities, which lead to a wide range of outputs, nearly all of them still suffer from a lack of clinical as well as external validation, standardization, and proper RCT to prove their superiority over medical service providers. Moreover, recent studies also indicate a continuous and competitive evolution in terms of their software architecture, from black-box to more interpretable types, due to the ethical advantages and technical feasibility of the latter [64]. Besides all the excitement and promises that AI-based tools can bring to infertility diagnosis and treatment, there also exist several important challenges that should be resolved to maximize their potential benefits in the area of reproductive medicine [77].

### 7.1. Concerns Regarding Data Quality, Integrity, and Safety

AI can delineate multilevel relationships of variables in a large amount of biological and medical data using complex algorithms and use these insights to create successful prediction models that can assist the clinical diagnosis as well as treatment steps. To achieve such a goal, the quality, integrity, and safety of the biological and medical data are of utmost importance. Although there has recently been considerable excitement and productivity regarding AI-based non-invasive tests and prediction algorithms in the field of reproductive medicine, nearly all of them suffer from important issues such as data heterogeneity, limited datasets, and different forms of bias. Before setting up AI-based prediction systems, it must be ensured that the data used to train these models is comprehensive, accurate, and representative of the target population [78]. Many existing datasets, such as the UK’s Human Fertilisation and Embryology Authority database, while extensive, lack crucial details like body mass index, lifestyle factors, and several key assessments of sperm, oocyte, and embryo quality [79]. This can limit the ability of AI algorithms to make reliable predictions from such big databases and generalize the predictions to diverse populations.

It is important to note that bias can occur in various stages, such as data collection, labeling, model training, or deployment of the algorithm. One of the primary sources of such bias is data collection. If the training data are imbalanced or the model architecture is not properly designed to account for diverse inputs, outputs may become biased. Also, algorithmic biases can arise from optimization techniques that favor majority group predictions over minority groups. The algorithm can perform well during the training, but a bias can still occur upon its deployment in real-world cases. It is therefore very important that the system should be tested with diverse inputs or monitored for bias after deployment to prevent unintended discriminations or exclusions.

Beyond the content, the integrity of the data is also of paramount importance. Establishing rigorous data quality standards and validation procedures is essential to reduce variability and promote collaboration across the field. Adherence to regulatory frameworks like IEC 62304 can help ensure the development of robust, clinically validated AI models that meet the necessary safety and efficacy requirements [80]. Comprehensive data protection measures, such as those outlined in the General Data Protection Regulation in Europe, must also be implemented to safeguard patient privacy and prevent unauthorized access or misuse of information. 

### 7.2. Relative Complexity of Female Infertility

Developing personalized approaches for infertility diagnosis and treatment is currently limited due to the level of our understanding regarding the pathophysiology of the condition. Still, in about 10–25% of cases, the condition falls into the “unexplained infertility” category. Furthermore, there can also be a “hidden” condition in a considerable number of couples who are diagnosed via classical approaches. Since female infertility is influenced by numerous factors, including hormonal, genetic, and environmental components that are still unknown, an AI-based algorithm may find it difficult to comprehensively evaluate and create a correct diagnosis with a limited amount of variables and data. AI algorithms require large, high-quality datasets to discern patterns and make accurate predictions; however, the heterogeneity inherent in female infertility presents a substantial obstacle to data acquisition and standardization. The intricate relationships between various clinical parameters, such as hormone levels, imaging results, and patient history, further demand sophisticated AI models capable of capturing non-linear dependencies and contextual nuances. 

### 7.3. Technical, Operational, and Financial Challenges/Limitations

The integration of AI-based tools into clinical workflows also presents a unique set of challenges that must be carefully navigated concerning technical, operational, and financial aspects [81]. Their integration into the clinical setting requires careful reorganization, training, and skill management of the medical and administrative staff as well as their role in the decision-making process. The development and deployment of AI technologies necessitate a robust technological infrastructure, which may be lacking in many regions, especially those with limited resources.

Operational challenges stem from the complexities of integrating AI tools into existing clinical workflows and ensuring seamless collaboration between AI systems and healthcare professionals. The successful translation of AI systems into clinical practice hinges on demonstrating their clinical usefulness and trustworthiness. Establishing trust in AI-driven diagnoses and treatment recommendations requires rigorous validation studies and transparent explanations of how AI algorithms arrive at their conclusions. Moreover, there is a critical need for standardized evaluation metrics and regulatory frameworks to ensure the safety and efficacy of AI-based diagnostic tools [79].

It is necessary to note that effective use of AI-based tools necessitates a complete digital transformation in the clinic where the required data can be generated, acquired, and processed in real time by the trained staff. Such an infrastructure comes with technological instrumentation and maintenance-related costs, data storage, backup, and security-related costs, costs for the regular training of the personnel, and HR costs for data scientists and other non-medical personnel to maintain the acquired/generated data safely. The costs of deploying AI solutions, including hardware, software, and training, can be prohibitive for many clinics and hospitals, particularly in resource-constrained settings.

### 7.4. Regulatory and Ethical Concerns

Regarding the integration of AI in the field of infertility diagnosis, a critical aspect to consider is the issue of dynamic information and consent. AI-based systems in reproductive medicine may continuously gather and process large volumes of patient data, including sensitive genetic and reproductive information. This raises questions about the extent to which patients can maintain informed consent and control over the use of their data, particularly as the algorithms and data processing evolve. To maintain the highest ethical standards, clinicians must ensure that consent processes explicitly address the involvement of AI, its limitations, and the nature of the data used to train such models. Consent should be an ongoing dialogue, not a one-time event, particularly when AI systems evolve or integrate real-time patient feedback. Moreover, patients should be given the option to opt out of AI-influenced components of their care without fear of compromised treatment quality.

Also, ownership and accountability for the performance and potential biases of these systems must be carefully established to ensure trust and accountability. The use of AI in clinical decision making raises fundamental questions about accountability. When AI systems make recommendations, the lines of accountability can become blurred between software developers, clinicians, and the institutions using these tools. Based on our current level of knowledge and limited experience, the best approach to address this challenge would be to position it as a decision-support tool rather than a decision-making authority. Clinicians must retain ultimate responsibility for patient care and be equipped to understand, interpret, and challenge AI-generated insights. Regulatory frameworks should mandate transparency in algorithmic logic and decision pathways, with clearly defined audit trails to ensure that decisions can be reviewed and evaluated retrospectively.

Privacy is also a significant concern. AI-based tools in reproductive medicine may have access to highly sensitive personal information, and the potential for data breaches or unauthorized access is a serious risk. The use of AI algorithms in areas such as fertility diagnosis, treatment, genetic screening, etc., also raises concerns about the possibility of discrimination, particularly against marginalized or vulnerable populations. To properly handle these concerns, comprehensive ethical and regulatory frameworks must be developed and implemented. Regulatory bodies should establish clear guidelines for the development, testing, and deployment of AI-based technologies in reproductive medicine, ensuring that they adhere to principles of transparency, accountability, and non-discrimination [82]. Addressing these challenges will require a multifaceted approach, involving close collaboration between technology providers, clinicians, data scientists, ethicists, and regulatory bodies. By prioritizing the quality and integrity of biological and medical data, the field of reproductive medicine can harness the full potential of AI to provide more personalized, equitable, and effective care for patients [83,84,85].

## 8. Future Directions

Based on the status of AI and AI-based technologies and their involvement in reproductive medicine, there is no doubt that their contribution to female infertility diagnosis and treatment will continuously be expanded. The authors of this article expect that this expansion will be towards several critical directions, such as:

- Integration and analysis of multi-omics and real-time data by AI in daily practice: AI systems are anticipated to increasingly combine genomic, proteomic, and metabolomic data with real-time physiological monitoring of reproductive health [86,87]. This integration could enable better counseling and more precise and personalized diagnostics and treatment recommendations, addressing the multifactorial nature of infertility.
- AI-augmented ART: Future AI models are likely to enhance embryo selection through advanced imaging and non-invasive genetic screening techniques. Algorithms will predict not only implantation success but also long-term health outcomes, optimizing patient care beyond conception [62,88].
- Explainable AI (XAI): As AI adoption grows, the demand for transparent and interpretable AI models will rise. XAI tools will help clinicians understand AI predictions, ensuring trust and accountability in critical reproductive health decisions [89].
- Global data standardization and federated learning: The development of global consortia and the adoption of federated learning approaches will improve data sharing while maintaining patient privacy. This collaboration will refine AI algorithms and expand their applicability across diverse populations.
- Remote and wearable technologies: AI integration with wearable devices will facilitate continuous monitoring of ovulatory cycles, hormonal levels, and other reproductive parameters that can be monitored non-invasively. These innovations will empower patients with actionable insights, bridging gaps in access to fertility care [34].

## 9. Conclusions

Current AI applications in female infertility diagnosis extend to areas such as predictive modeling of ovulatory disorders, automated interpretation of medical imaging (e.g., ultrasound and hysterosalpingography), and the identification of genetic markers linked to reproductive health. These AI-driven solutions can enable earlier and more precise identification of underlying causes of infertility, allowing for tailored treatment plans that align with individual patient profiles. Additionally, once the accurate diagnosis is achieved, integrating this information into AI-based management tools can also efficiently assist in monitoring treatment progress and optimizing ART by predicting success rates and recommending personalized interventions.

Besides these promising advantages, the integration of AI in infertility diagnosis nowadays faces several challenges, including the need for large, high-quality datasets, lack of clinical validation of the developed algorithms by independent external institutions, concerns related to ethical considerations, such as data privacy, consenting, and accountability, and the requirement for interdisciplinary collaboration between medical professionals, data scientists, and regulatory bodies. Furthermore, the black-box nature of some AI models presents transparency challenges, which may also impact clinical trust and adoption.

Despite these challenges, research, development, validation, and integration of AI algorithms or AI-based digital applications in the field of reproductive medicine are exponentially increasing. Besides aiming to improve and successfully perform multicenter clinical validation studies for the existing tools, future research should involve standardization of data acquisition processes together with the creation of interpretable AI tools that can be utilized globally. As in other branches of medicine, reproductive medicine has already started to experience AI-driven digital transformation worldwide, which is expected to reshape our diagnostic and therapeutic practices and open numerous new avenues as well as hurdles in the field of female infertility diagnosis and management.
